# Designing Technology for Aging and Disability

**DOI:** 10.1093/geroni/igaa057.3175

**Published:** 2020-12-16

**Authors:** Shelia Cotten

## Abstract

Technology has massive potential to improve the lives of older adults in terms of their health, wellbeing, quality of life, and independence. However, benefits will not be realized unless these technologies are designed considering the needs, abilities, and attitudes of the diverse population of older adults. This is especially true when we consider older adults experiencing physical, sensory, or cognitive impairments that influence their ability to adopt and use technology-based solutions. This symposium highlights a variety of approaches to using technology to support older adults living with disability, and important design considerations. The first talk will highlight the important role technology can play in helping persons with cognitive and /or physical impairment in the workplace. The next talk outlines a framework and methodology designed to provide older adults with hearing or visual impairments the capability to use and adapt digital health tools. This is followed by a discussion of a research agenda to use technology to help older adults experiencing disability as a result of cognitive impairment participate in their community. Then, there will be a discussion of how digital health technologies, when considering their unique needs and abilities, can support older adults with cognitive impairment and dementia. The final talk focuses on the intersection of technology, cognitive impairment, and leisure, and explores engagement with digital games by older adults with and without dementia. Common themes that emerge and future directions will be highlighted.

